# Effects of carbon sources on the enrichment of halophilic polyhydroxyalkanoate-storing mixed microbial culture in an aerobic dynamic feeding process

**DOI:** 10.1038/srep30766

**Published:** 2016-08-03

**Authors:** You-Wei Cui, Hong-Yu Zhang, Peng-Fei Lu, Yong-Zhen Peng

**Affiliations:** 1 Beijing University of Technology, College of Energy and Environmental Engineering, 100 Pingleyuan, Chaoyang District, Beijing 100124, China

## Abstract

Microbial polyhydroxyalkanoate (PHA) production serves as a substitute for petroleum-based plastics. Enriching mixed microbial cultures (MMCs) with the capacity to store PHA is a key precursor for low-cost PHA production. This study investigated the impact of carbon types on enrichment outcomes. Three MMCs were separately fed by acetate sodium, glucose, and starch as an enriching carbon source, and were exposed to long-term aerobic dynamic feeding (ADF) periods. The PHA production capacity, kinetics and stoichiometry of the enrichments, the PHA composition, and the microbial diversity and community composition were explored to determine carbon and enrichment correlations. After 350-cycle enriching periods under feast-famine (F-F) regimes, the MMCs enriched by acetate sodium and glucose contained a maximum PHA content of 64.7% and 60.5% cell dry weight (CDW). The starch-enriched MMC only had 27.3% CDW of PHA. High-throughput sequencing revealed that non-PHA bacteria survived alongside PHA storing bacteria, even under severe F-F selective pressure. Genus of *Pseudomonas* and *Stappia* were the possible PHA accumulating bacteria in acetate-enriched MMC. Genus of *Oceanicella*, *Piscicoccus* and *Vibrio* were found as PHA accumulating bacteria in glucose-enriched MMC. *Vibrio* genus was the only PHA accumulating bacteria in starch-enriched MMC. The community diversity and composition were regulated by the substrate types.

Microbial polyhydroxyalkanoate (PHA) production has attracted research and development interests due to its biodegradability and its potential to serve as a substitute for petroleum-based plastics^1^. The possibility of producing PHA at a low cost has motivated advancements in using mixed microbial culture (MMC) as a production method. MMC biotechnology may reduce production costs because it has: (1) no sterilization requirements, (2) an adaptive capacity due to microbial diversity, and (3) the possibility of using renewable substrates^2^.

The PHA production method using MMC has two steps. First, substrates are used to enrich the culture. Second, the culture, highly enriched in PHA-producing microorganisms, is fed with substrate for PHA storage. Enriching the functional bacteria from the inoculum constitutes the first step and the most important component of two-step PHA production by MMC. Enriching functional MMC commonly adopts an aerobic dynamic feeding (ADF) strategy. The ADF process is often referred to as the feast-famine (F-F) process, where microorganisms are cultivated in a selective environment that alternates the presence and absence of external substrate^3^. The ratio of feast time to famine time (F/F) is the selective pressure that regulates the competitive activity of PHA storing and non-PHA storing bacteria. Because the PHA-accumulating bacteria stores substrate faster during the feast period, this type of bacteria establishes its competitive growth advantage over non-storing organisms during the famine period^4^.

Several researchers have successfully selected cultures with high-content PHA production capacities, by imposing F-F conditions in a sequencing batch reactor (SBR). Using an ADF strategy, one research team collected activated sludge from the ‘Roma Nord’ full-scale plant and enriched it with PHA content higher than 50% (on a COD basis) by feeding the activated sludge with feedstock composed of acetic, lactic, and propionic acids^5^,^6^. Johnson *et al*. operated an acetate-enriched SBR with F-F cycles of 12 h to enrich a mixed culture of PHA producers^1^. Another study achieved the development of MMCs capable of producing PHAs by periodically feeding with nonanoic acid in a SBR in a municipal wastewater treatment plant in Daejeon, Korea; this allowed the accumulation of maximum PHA content of 48.6% dry cell weight (CDW)^7^.

In these studies described above, the single volatile fatty acid (VFA) or mixed VFA produced by acidogenic fermentation was applied as the enrichment substrate. These studies show that VFA can be directly converted into PHA, facilitating PHA functional bacteria selection by providing a competitive advantage to PHA-storing organisms^8^. Following these achievements, even the enrichment model was developed only using VFAs as substrate^9^.

To reduce PHA production cost, significant effort has been invested in using inexpensive carbon substrates and developing more efficient fermentation processes^10^. To this end, a three-step strategy for polymers production has been used for waste-based PHA production, based on a two-step process. The pretreatment process converts the wastewater or wastes into VFAs, prior to the two steps of enrichment and production. The fermented product from the pretreatment process is used in the subsequent steps of enriching PHA-storing bacteria and producing PHA. Applying this three-step strategy, many researchers have tested renewable carbon sources for their use in PHA production, including of waste starch, molasses, lignocellulosic waste, whey from dairy industry, glycerol from biodiesel production, slaughterhouse waste, and waste oils^11^,^12^,^13^,^14^.

Although the three-step process makes waste-based PHA production possible, the elongated PHA production line significantly increases production cost compared to the two-step process. If the readily biodegradable chemical oxygen demand (COD), even biorefractory COD in the wastewater, could be used directly to enrich PHA storing bacteria, and subsequently for biopolymer production units. Thus, some wastewater could be directly used for PHA production in the two-step process. This would make waste-based PHA production more attractive.

Most recently, a bacterial community with high biopolymer production capacity was successfully enriched using glycerol, a non-fermented substrate (i.e. non-VFA substrate)^15^. The study achieved high PHA producing capacities in the community using glycerol, glucose, lactate, and acetate as substrate. This study demonstrated that some non-VFA substrate could be applied to develop a PHA-storing community. That pointed to the possibility of using waste as a substrate to enrich PHA-storing MMC and produce polymer in the two-step process. However, due to limited research, three aspects remain unclear: (1) what kind of non-VFA matter can enrich PHA-storing MMC, (2) to what extent the substrate spectrum affects enrichment function and composition, and (3) what the composition of the produced PHA is.

Halophiles are receiving more and more attention as novel candidates for producing PHA^16^. Due to their dependency on salts for growth, halophilic bacteria are best as part of an open unsterile fermentation process, reducing the expenses of aseptic operations^17^. Applying halophilic cultures to produce PHA has also been suggested to facilitate the recovery of produced PHA, because halophiles can be lysed in distilled water, reducing the large quantities of organic solvents required to recover PHA^18^. Using a halophilic MMC could strengthen these advantages, because MMCs may reduce costs. As such, using halophilic MMC cultures provides a more sustainable and economical alternative for producing PHA, because they use wastewater as substrates, and require less expensive and unsterilized equipment^19^.

This study explored the effect of three kinds of substrate (acetate, glucose and starch), applied during the enrichment process under a F-F regime. The three substrates have incrementally more complex chemical structures, and were separately applied to enrich PHA storage MMC. The study used identical estuarine sediments, inoculated to enrich a halophilic PHA storing community for the first time. The differences between the halophilic enrichments were explored by comparing the PHA production capability and the PHA composition. The kinetic dynamics were analyzed alongside the bacterial community information to explore the mechanism of the substrate effects on these halophilic enrichments. Special attention was placed on investigating the consistency of PHA production capacity and the enrichment community as a function of the substrate spectrum.

## Results

### PHA storing ability of enrichments using three kinds of substrates

**Figure 1:**
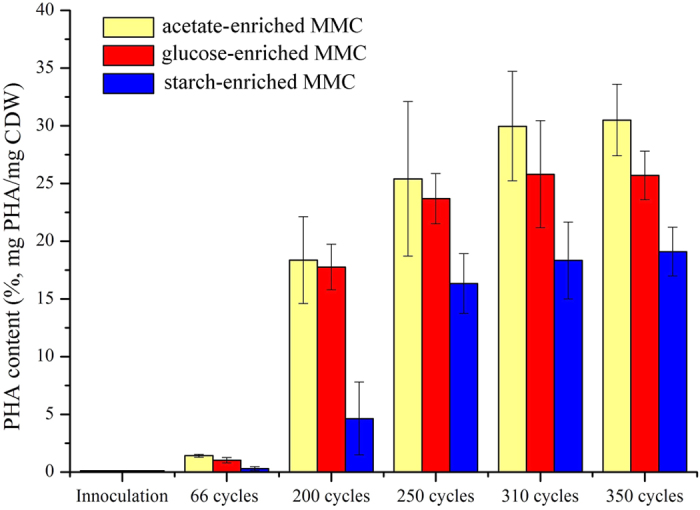
Variations in the maximum PHA content in the three MMCs during the selection period operational cycles. The error bars represent the standard deviations (SD).

All SBRs were operated identically with an enrichment time of 350 continuous cycles (70 SRTs). The steady state could be judged by examining the variation in maximum PHA content during operational cycles, along with enrichment time (Fig. 1). In the 310th cycle, the maximum PHA content in the three MMCs increased from 0.12% of CDW in the initial inoculum up to 30.5%, 25.5% and 18.2% of CDW for acetate sodium, glucose, or starch, respectively. The maximum PHA content remained relatively stable after the 310th cycle, with no more than 4.7% standard deviation in three consecutive cycles. This stability suggests that the three MMCs have reached the steady state.

**Figure 2:**
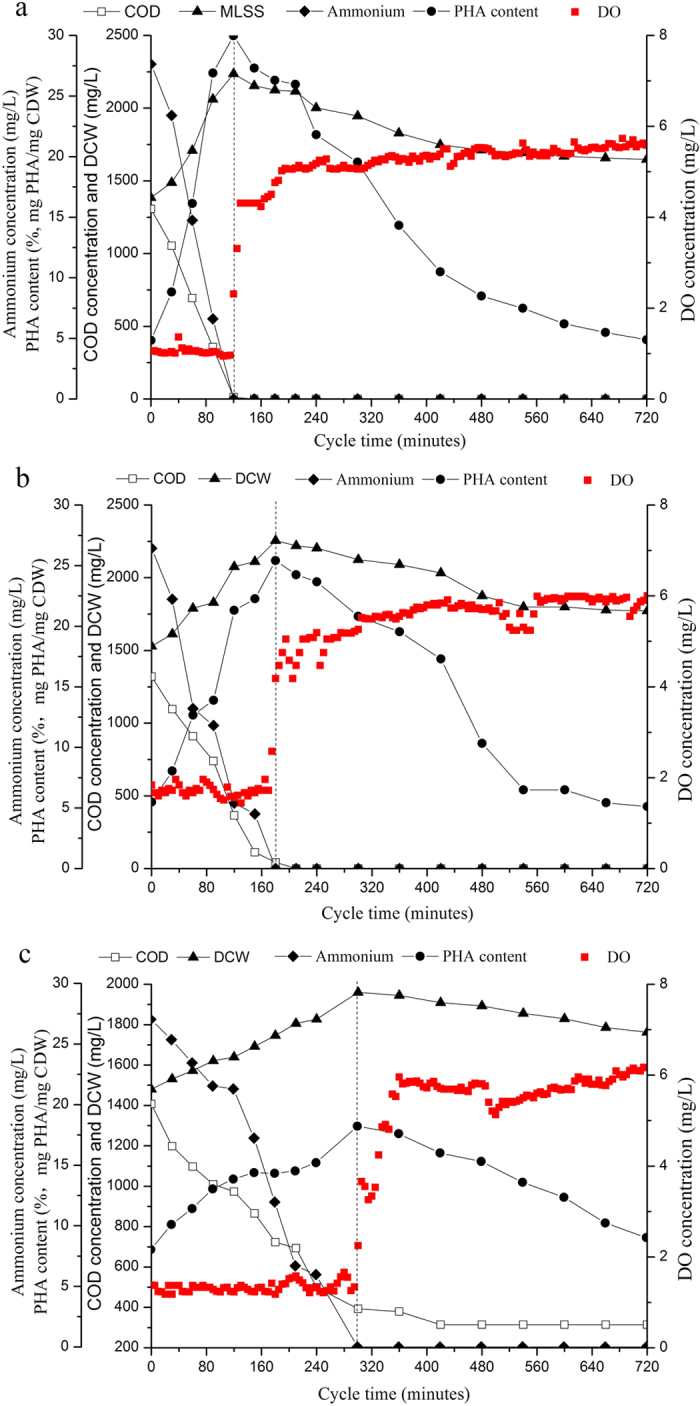
Data from a typical cycle, monitored after the 310th cycle, indicating COD and ammonium utilization dynamics, PHA content, and DO evolution. (**a**) acetate-enriched MMC, (**b**) glucose-enriched MMC, (**c**) starch-enriched MMC.

The F-F cycles of three enrichments in the steady state were monitored. Figure 2 shows biomass and PHA production profiles and substrate and nitrogen consumption during the batch studies. The PHA cell content and the biomass increased as COD and ammonium concentrations decreased in the three enrichments. The results indicated that the PHA synthesis and biomass proliferation process occurred simultaneously with the presence of COD and ammonium nitrogen (called the feast period). After COD and ammonium were depleted (called the famine period), the PHA content and the biomass concentration immediately decreased. During the famine period, the MMC began to consume the endogenously stored PHA, because there was no available carbon substrate.

The F-F cycles showed the similar trends, except for the completion time and the consumption amount of the external substrate. In the acetate-enriched MMC, after 120 (±8) min, 1306 mg COD∙L^−1^ acetate and 27.6 mg N∙L^−1^ ammonia was consumed, with bacterial proliferation of 740 mg CDW∙L^−1^ and a PHA storage of 30% of CDW (Fig. 2a). Glucose-enriched MMC showed similar characteristics as the acetate-enriched MMC (Fig. 2b). In the starch-enriched MMC, within 300 (±10) min, 27 mg N∙L^−1^ ammonia was completely consumed, while 393 mg∙L^−1^ COD remained (Fig. 2c). These results indicate a difference in substrate utilization rates across the three enrichments.

The dissolve oxygen (DO) curve changed regularly across the full cycle, with some characteristic indicators. As the exogenous substrates were rapidly consumed, DO remained at a steady low level, as long as there was a constant air supply rate. When the exogenous organic matter was completely depleted, the DO quickly increased to a high plateau, because the oxygen consumption rate was greatly reduced. The DO profiles in the three systems varied in an identical way (Fig. 2), demonstrating good reproducibility. This cyclical variation in DO is consistent with other research^20^, and has been used to mark the boundary of the feast and famine period. This study applied this same practice, using DO to monitor the boundary of the feast and famine period during the pulse-fed tests of maximum PHA production.

**Figure 3:**
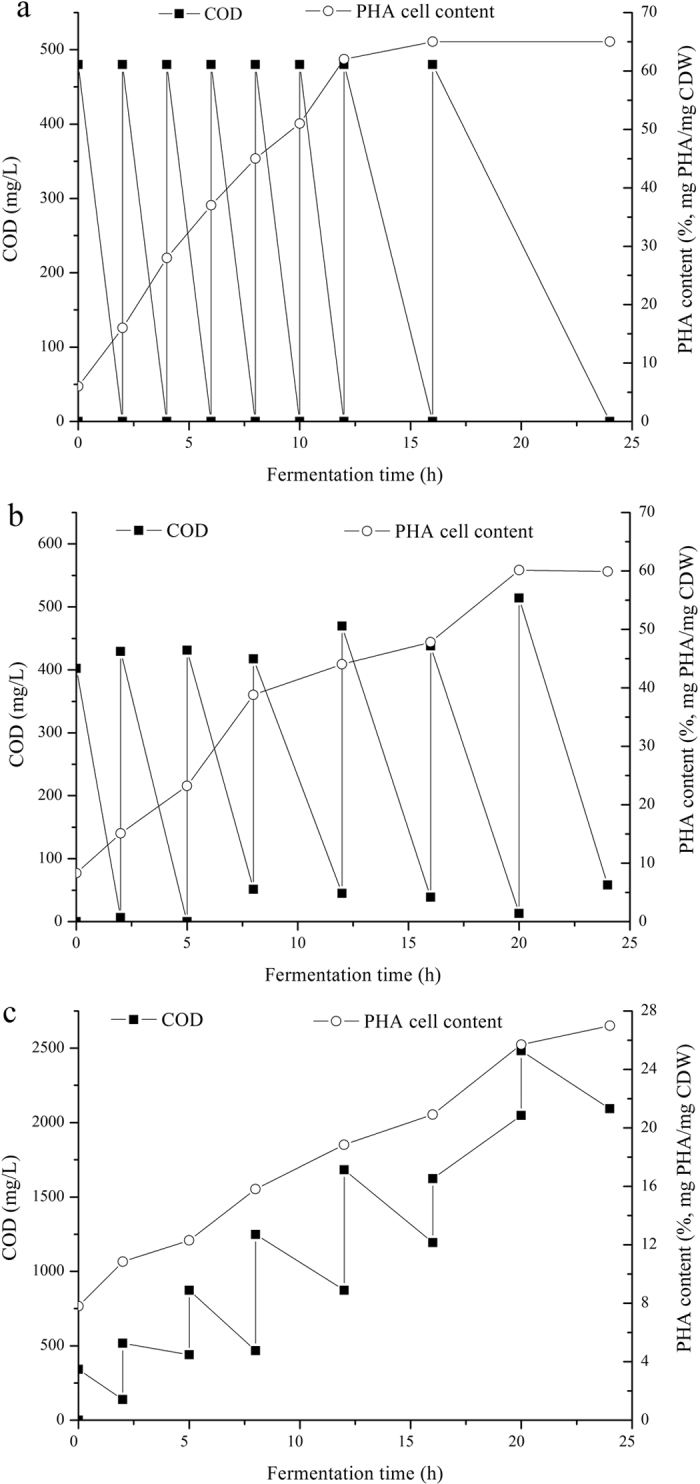
The maximum PHA production capacity in the fermentation stage, tested using pulse-wise feeding strategies. (**a**) acetate-enriched MMC, (**b**) glucose-enriched MMC, (**c**) starch-enriched MMC.

The maximum PHA storage capacity of the enrichments was further determined using pulse-wise feeding strategies to avoid potential substrate inhibition. In the pulse-wise feeding experiments, all the enrichments had maximized PHA storage after the seventh supply of substrate. The MMCs enriched by acetate sodium and glucose held a maximum PHA content of 64.7% and 60.5% CDW, respectively. The MMC enriched with starch resulted in only 27.3% CDW PHA (Fig. 3). These data suggest that MMCs enriched by chemically complicated carbon sources (such as starch) could lead to low PHA storage capacity. In contrast, chemically simple carbons (such as glucose) may be better able to enrich high PHA storing MMCs. This result is similar to other studies using freshwater MMC. In some studies, MMC enriched with acetate acid showed PHA storage capability of 50% CDW content^1^,^21^. In studies where more complex substrates were used to produce PHA, such as molasses, whey, cellulose, hemicellulose, sucrose and palm oil, the polymer cell content has been consistently lower compared with the studies where simple carbon sources were used^22^.

The low PHA accumulation is likely due to the low-speed use of starch. The PHA content in the enrichment periods and during the pulse-fed experiments was almost equal, indicating that substrate concentration did not limit PHA synthesis. The starch-enriched MMC only used part of the starch each time substrate was added, resulting in COD accumulation in the mixed liquids (Fig. 3c). Sufficient time for biodegradation was provided; however, a fraction of the starch remained, based on the COD analysis (Fig. 2c). This outcome was seen during every enrichment cycle, demonstrating that the further bioconversion of starch was limited by its relatively complex chemical structure. In contrast, the acetate-enriched and glucose-enriched MMCs used almost all the supplied COD. This leads to the conclusion that carbon biodegradability significantly impacts enrichment outcomes.

### Kinetics and stoichiometry of enrichments

The kinetics and stoichiometry of the three enrichments was determined using three continuous cycle dynamic curves after 310 operational cycles (Table 1). The fastest specific substrate uptake rate (*q*_s_) was registered as 0.461 ± 0.022 mg∙mg VSS^−1^∙h^−1^ in the acetate-enriched MMC. Carbon uptake supported fast biomass growth (*μ*_max_, 0.082 ± 0.003 h^−1^) and fast PHA storage (*q*_PHA_, 0.292 ± 0.015 mg∙mg VSS^−1^∙h^−1^). Martins *et al*. found that a freshwater MMC accumulated PHA with a similar *q*_s_ and *q*_PHA_ as this study found, using acetate as the only carbon^23^. In our study, the *μ*_max_ and *q*_*s*_ of glucose-enriched MMC and the acetate-enriched MMC were very similar, while the *q*_*PHA*_ of the starch-enriched MMC was only half of that of the acetate-enriched MMCs (Table 1). The *q*_PHA_ of the starch-enriched MMC was only 0.032 ± 0.031 mg∙mg VSS^−1^∙h^−1^, approximately one-quarter that of acetate-enriched MMC (Table 1). This result further supports the assumption that *μ*_max_ and *q*_s_ are highly dependent on organic matter biodegradation. In this study, *μ*_max_ and *q*_s_ were differentiated based on the refractory nature of starch, compared to the readily biodegradable acetate and glucose.

It is likely that *q*_PHA_ was driven by the complexity of the carbon chemical structure, rather than the biodegradability. This is because in nature, the enrichment *q*_PHA_ can be regulated by the different biological PHA synthesis pathways of differently structured carbons. Acetate is considered a “PHA metabolism-related” source, structurally identical to PHA monomers. This leads to direct PHA biosynthesis from the *β*-oxidation of fatty acid pathways^24^. Glucose is a “PHA metabolism-unrelated” source, and can be synthesized into PHA through six pathways^3^. These pathways generally rely on pyruvate production, which is first metabolized through glycolysis *in vivo*. As such, the glycolysis pathway regulates the PHA synthesis rate. According to the kinetics of the starch-enriched MMC, the limited availability of PHA precursors derived from the biological conversion of starch may have resulted in the low *q*_PHA_. Starch can be converted into glucose through a complicated metabolic process, with the cooperation of a microbial community rather than a pure culture. Therefore, each MMC’s PHA synthesis rate depended on the rate of the feedstock conversion into PHA precursors.

Table 1 lists the average observed storage yield (*Y*_PHA/S_) and average observed growth yield (*Y*_X/S_) values of the three enrichments, indicating the carbon allocation for the two energy-demanding processes. For the acetate-enriched MMC acetate, 60% of COD was used to form PHA, while 24% of COD was used to grow active biomass. For the glucose-enriched MMC, 54% of glucose was used to form PHA and 24% of glucose was used for biomass growth. The starch-enriched MMC differed from the acetate-enriched and glucose-enriched MMCs: the carbon transformation into PHA was lower, at 30%, which was equal to the amount transformed into active biomass.

These data suggest that more carbon was directed into PHA formation when simple carbons were used; more carbon was directed to biomass growth when a more chemically complex carbon was used. When compared with other research using freshwater MMCs with acetate and mixed VFA^23^, the *Y*_PHA/S_ values of glucose-enriched and acetate-enriched MMCs in this study were higher; further, the *Y*_PHA/S_ value of the starch-enriched MMC in this study was even higher than the freshwater glycerol-enriched MMC^25^. These data suggest that the halophilic MMC used carbon to form PHA more efficiently than freshwater MMCs.

During the famine period, microbes survive by using endogenous matter, quantified using decay rate (*k*_d_). High PHA content in the biomass provides electron donors for endogenous respiration, slowing *k*_d_. Because *k*_d_ likely reflects the PHA accumulating in microbial cells, it is a key parameter reflecting enrichment outcome. The starch-enriched MMC had a *k*_d_, nearly twice as fast as the acetate-enriched and glucose-enriched MMCs. To account for these *Y*_PHA/S_ and *k*_d_ levels, there must be significantly higher numbers of PHA-storing organisms in the acetate-enriched and glucose-enriched MMCs than in the starch-enriched MMC. These findings strongly support the hypothesis that the substrate species may account for the differences in PHA-storing population in MMCs.

The apparent half-saturation constants (*K*_s_) for organic matter uptake differ between the three enrichments. The acetate-enriched MMC had the largest apparent *K*_s_, while the starch-enriched MMC had the smallest *K*_s_. Small *K*_s_ values are usually associated with larger affinities, which indicates the microbe’s ability to collect a substrate through permeases or initial enzymes. These data suggested that acetate-enriched MMC predominated eutrophic environments, while starch-enriched PHA-storing organisms had a competitive advantage over non PHA-storing organisms in the low-concentration substrate environments. The results supported the conclusion that low PHA accumulation in the starch-enriched MMC was induced by the low availability of VFA converted from starch.

### Components of PHA produced by three MMCs

The PHA produced by the three enrichments shows the different compositions. Pure PHB polymer was produced by the acetate-enriched MMC; the hydroxyvalerate (HV) monomer of PHA was synthesized in the glucose-enriched (with 5–7% CDW) and starch-enriched MMCs (with 10–15% CDW). A larger HV percentage was synthesized in the starch-enriched MMC than in the glucose-enriched MMC. The PHA components depended on the feedstock composition and the PHA-storing organism^26^. Propionate is an accepted building block to incorporate HV units in PHAs, because the propionate-dependent pathway regulates polyhydroxyvalerate (PHV) synthesis^27^. In this respect, the increasing HV fraction in PHA from enriched unrelated carbon sources (glucose and starch) suggests that more propionate may have been produced in the starch-enriched MMC.

### Bacterial diversity analysis based on OTUs

Tackling community diversity is important, because biodiversity can influence MMC functionality. Given this, we further investigated the diversity of the three enrichments by applying Miseq-pyrosequencing, with their original seed as the control. After rigorous quality control, 73099 reads remained, with a read length of more than 400–480 bp. This accounted for more than 99.9% in all sequences (Fig. S1).

Rarefaction analysis was completed at the operational taxonomic unit (OTU) level. The rarefaction curves plateaued in phases in all samples, indicating substantial and complete sampling for bacterial richness (Fig. S2). The sequencing data were grouped into OTUs at a 3% cut-off level, resulting in a total of 275 different OTUs. As the Venn diagram shows, only 3 OTUs were shared between four samples (Fig. S3). This suggests that only a few bacteria could survive in both the natural and the engineered niches.

A Simpson analysis is considered to be a robust measure of diversity. This analysis showed that the inoculated seed had apparent diversity, with Simpson values close to 1 (Table 2). The seeded estuarine sludge had the highest diversity; this conclusion was supported by the computation of richness using the Chao1 estimator. Because environmental factors shape ecological diversity, it is not surprising that the estuarine sediments seeded as inoculum had the highest diversity and richness. Many variables, such as salinity, nitrogen, pH, oxygen, sulfide, and organic loading, have steep physical-chemical gradients in the estuary; some of these correlate with microbial community shifts^28^. Because of the steep gradients, these estuary habitats offer many possibilities for highly diverse microbial communities to form.

In contrast to natural niches, the engineered SBRs were more controlled and stable, with the same operational parameters and only the single substrates. This lack of environmental variability dramatically lowered the ecological diversity in the engineered reactors; however, the three enrichments did generate different diversity and richness levels. The glucose-enriched MMC had the highest diversity and richness of the three, likely associated with the growth of organisms better suited to that feedstock. The lowest diversity and richness was seen in the starch-enriched MMC. The diversity in the engineered enrichments suggests that applying chemically different carbon sources results in different forms of bacterial diversity, even under well-controlled conditions.

### Community composition

**Figure 4:**
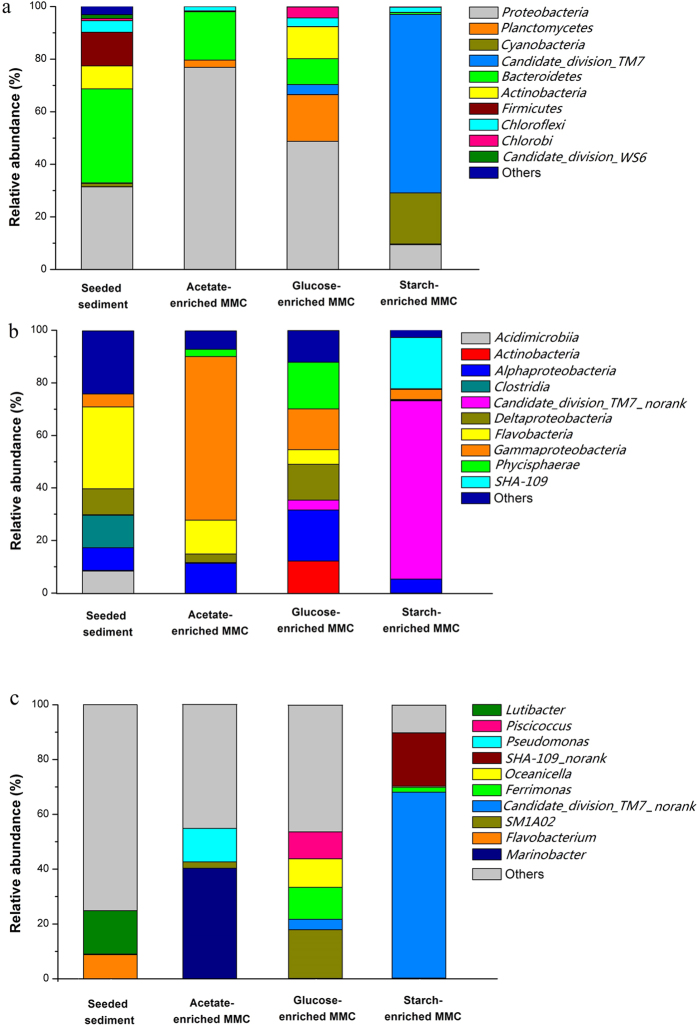
Relative abundance of bacteria affiliated with phyla (**a**), class (**b**) and genus (**c**) in seeded sediments and the three enrichments.

Knowing which organisms are involved in PHA-producing communities is critical to understanding the functionality of enriched MMCs; however, little is known about the community composition selected under the pressure of ADF. To study this, we investigated the community composition of the three enrichments. Figure 4 shows the top 10 phyla, classes and genera (on a % OTU basis), as well as the combined total for the remaining phyla/classes/genera (classed as “others”). Bacteria belonging to *Bacteroidetes* and *Proteobacteria* phylum were abundant, with 35.7% and 31.4% relative abundance in the original seed, respectively. After being highly enriched, the bacterial communities in the acetate, glucose, and starch-enriched MMC dwindled to 8, 10, and 8 phyla, respectively. No other phylum appeared after the original seed screening. After enrichment, bacteria affiliated with the *Proteobacteria* phylum dominated the acetate-enriched MMC, with a relative abundance of 76.9%; the relative abundance of the *Bacteroidetes* phylum decreased to 18.4%. These results suggest that the bacteria in the *Proteobacteria* phylum had a stronger competitive preference for the acetate substrate than other bacteria. This also indicated the *Proteobacteria* was more likely to endure the famine phase, relying on its PHA-storing ability.

The bacterial community in the glucose-enriched MMC was mainly composed of the *Proteobacteria* phylum, with a relative abundance of 48.7%; *Planctomycetes* phylum, with a relative abundance of 17.7%; *Actinobacteria* phylum with a relative abundance of 12.1%; and *Bacteroidetes* phylum, with a relative abundance of 9.8%. In addition to *Proteobacteria*, some bacteria belonging to the *Actinobacteria* phylum also had PHA-storing ability. The starch-enriched MMC was largely dominated by the *Candidate_division_TM7* phylum, with a relative abundance of 67.8%. This phylum accounted for less than 0.2% in the original seed. Bacteria affiliated with the *Cyanobacteria* phylum was also enriched, with a relative abundance of 19.4%. The relative abundance of *Proteobacteria* and *Bacteroidetes* phylum subsequently dropped to below 9.4% and 1%, respectively.

Despite these different abundant levels, bacteria affiliated into *Gammaproteobacteria* class were found in all samples; within this class, more than 20 PHA-accumulating genera were found (Fig. 4b). The relative abundance of *Gammaproteobacteria* class (5.41%) in the original seed was augmented to levels of 62.83% in the acetate-enriched MMC and the 15.63% in glucose-enriched MMC, respectively. However, their abundance was only 3.85% in the starch-enriched MMC. Further, bacteria in the *Alphaproteobacteria* class were also observed in all samples, albeit with low abundance levels. A large proportion of PHA-storing bacteria including *Halomonas*, *Vibrionaceae* and *Pseudomonadaceae* belong to this class. Bacteria in *Phycisphaerae* class were enriched in the acetate-enriched MMC (2.8%) and the glucose-enriched MMC (17.7%). No previous studies have suggested that bacteria in the *Phycisphaerae* class are able to accumulate PHA. Bacteria in the *Candidate_division_TM7* phylum are also distributed widespread in soil and the activated sludge, but are also not known to accumulate PHA^29^.

On the phylogenetic taxa level of genus (Fig. 4c), genus of *Dechloromonas*, *Thauera*, *Rhodobacter*, *Pseudomonas*, and *Vibrio*, belonging to the *Proteobacteria* phylum, with relative abundance levels lower than 1%, have been reported to accumulate PHA in seed sludge^30^. Two genus belonged to *Betaproteobacteria*, two belonged to *Gammaproteobacteria* and one belonged to *Alphaproteobacteria*. When enriched with the acetate sodium, the relative abundance of *Pseudomonas* genus increased to 12.25%. *Marinobacter* genus was highly enriched, with a relative abundance of 40.43% as the dominant bacteria. Based on optimal salinity requirements, *Marinobacter* is a moderate halophile often found in sea sand and marine sediments, quickly proliferates in fatty acid substrates, and shows no ability to store PHA. The third highly-abundant group found in this study was the marine bacteria, *Stappia* genus (8.38%), which uses fatty acids to synthesis polymer^31^. This genus belongs to the *Alphaproteobacteria* class, which had the broad ability to store PHA. In summary, groups with the ability to store PHA made up 20.63% of the bacterial abundance in the acetate-enriched MMC.

In the glucose-enriched MMC, genus of *SM1A02*, *Ferrimonas*, *Oceanicella* and *Piscicoccus* dominated, with relative abundance levels of 17.7%,11.67%, 10.37% and 9.80%, respectively. *Oceanicella* is a moderate halophile with the potential ability to store PHA; it belongs to the same family with the known PHA-storing bacteria *Rhodobacter*. *Piscicoccus* also has the potential to store PHA because of the close phylogenetic relationships with the known PHA-storing genus *Kineosphaera*^32^. Other enriched PHA-storing bacteria include *Vibrio*, with a relative abundance of 2.29% in the glucose-enriched MMC. So, the abundance of the possible PHA storing groups account for 24.33% of the community of glucose-enrichment MMC.

The two unclassed genus affiliated with the phyla of *Candidate_division_TM7* and *Cyanobacteria* dominated the starch-enriched MMC, with relative abundance levels of 67.84% and 19.44%, respectively. These two dominant genus are not reported to have PHA storing ability. The only genus present in the starch-enriched MMC with PHA storage ability was *Vibrio* (1.96%).

## Discussion

In this study, we used estuarine sediment as inoculum to successfully enrich halophilic MMCs. The MMC enriched with acetate sodium as the sole carbon was able to store high PHA content, up to 64.7% of its cell weight. For the first time, this research demonstrated that it is feasible to enrich halophilic MMC to support PHA production from natural seed. Pyrosequencing revealed a wide phylogenetic diversity in the estuarine sediment (Table 2), directly supporting the enrichments. Although the study achieved success in using estuarine sediment as inoculum, it is unknown whether this approach can be easily extended to other halophilic inoculum from natural salty fields, where consortia vary tremendously. Community composition is determined by a number of factors, including different microenvironments with different physical and chemical properties. As a result, the community in salty fields could be very different, leading for the need for more study about how salty fields can provide the best seed sources to enrich halophilic PHA functional MMCs.

Several researchers have demonstrated how imposing F-F conditions in ADF process can successfully select cultures that have high PHA capacities^1^,^6^,^33^. In these studies, two forms of VFA were tested: pure VFA and mixed VFA produced from renewable feedstock by acidogenic fermentation. However, these studies did not explore whether the F-F regime remains effective when non-VFA carbon source is applied to the MMC enrichment.

**Figure 5:**
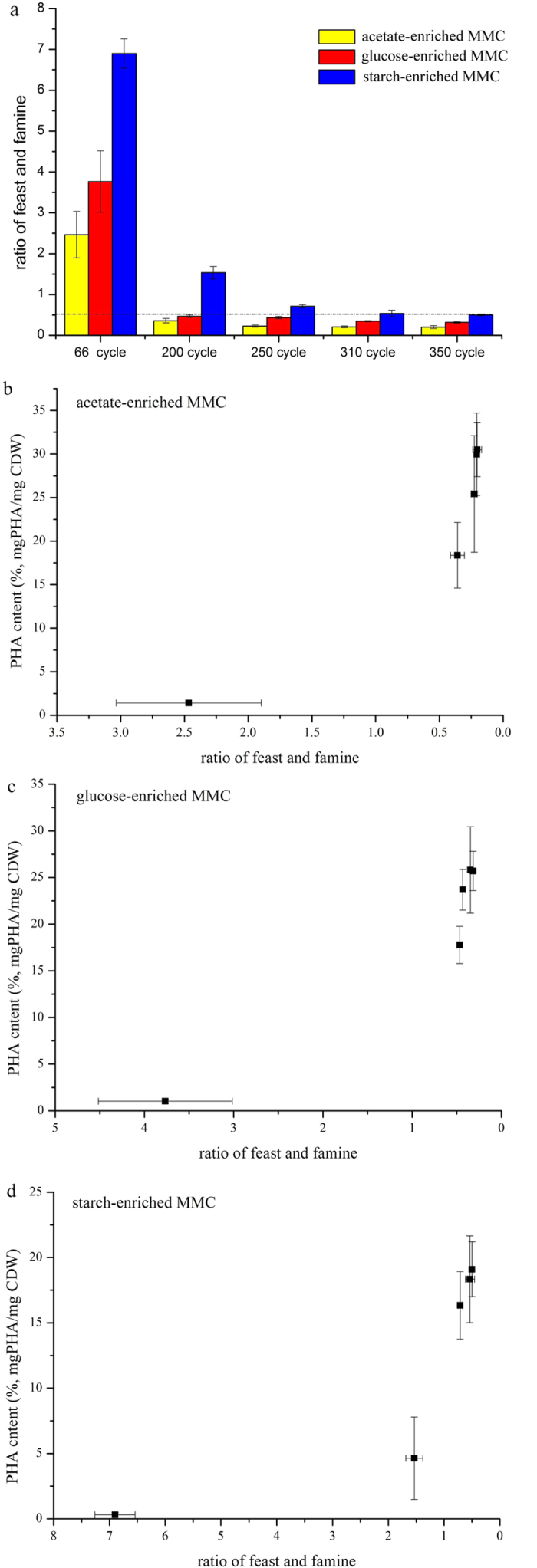
F/F ratio along with the operational time (**a**), the relationship between maximum PHA content with F/F ratios in the acetate-enriched MMC (**b**), the glucose-enriched MMC (**c**) and the starch-enriched MMC (**d**) during the enrichment periods.

In our study, three kinds of organic matter with different chemical structures were used to enrich PHA storing bacteria. All enrichments under the F-F regime heightened PHA storage capability (Fig. 3), and the F/F decreased continuously as enriching time progressed (Fig. 5a), reaching a constant values in the three systems after 310 days (i.e. acetate-enriched MMC reached 0.3, glucose-enriched MMC reached 0.35, and starch-enriched MMC reached 0.5). This indicates that the imposed selective pressure increased as selection time proceeded.

Further analysis of maximal PHA content and the imposed F/F during the enriching periods allowed to finding their relevance (Fig. 5b–d). The maximal PHA storage capacity of three enrichments increased with the imposed F/F deceased and the maximal PHA storage capacity almost kept stable when the F/F below 0.5. This data suggested that the F/F imposed on MMC will be decisive on the outcome of enrichment, independent of the applied carbon. Previous studies also reported that a F/F imposed on MMC less than 0.5 was a determining factor in enriching the freshwater culture with a PHA storage capability^1^. All the steady state F/F values in our study fell into this range, suggesting the F-F regime was effective for PHA-storing enrichment.

This study also revealed different PHA-storing capabilities of enrichments. The MMCs enriched by acetate sodium and glucose produced the most PHA, while starch-enriched MMC produced less (Fig. 3). This result is possibly due to two factors. First, the different F/F was imposed on the three systems (Fig. 5a). A stronger selective pressure imposed on the acetate-enriched MMC and glucose-enriched MMC could contribute to a high PHA storage capability.

Second, the PHA storage capability of the three MMCs could be induced by the availability of precursors supporting PHA synthesis. Acetate can be directly converted into PHA as the precursor, facilitating the selection of PHA functional bacteria by providing the competitive advantages for PHA-storing organisms. Glucose was the most readily biodegradable substrate, and can be transformed into pyruvate by the glycolysis pathway. As the refractory substrate, starch cannot be directly utilized. As such, PHA synthesis by the starch followed the same metabolic pathway originating from acetate or glucose; it depended on the bioconversion of starch into acetate or simple sugar. This availability of PHA precursors (acetate or pyruvate) was supposed to affect the growth of PHA storing bacteria under the F-F regime. The starch-enriched MMC had a low *q*_s_, indicating a limited bioconversion. The limited product of starch bioconversion could explain the low PHA capability in the starch-enriched MMC.

Although enrichment presented specific phenotypic behaviors supporting PHA storage, the underlying ecology and community was distinct. The pyrosequencing results of three MMCs showed wider phylogenetic diversity and a more complicated community than previously reported and demonstrated that non PHA-storing bacteria also survived under F-F regime. Three enrichments fed by different substrates created three communities of microorganisms that evolved well to perform a very specific function in a highly organized manner. The three enrichments showed a very district structure (Fig. 4). Principal components analysis (PCA) was used to identify physiological similarities among the three enrichments (Fig. S4) and assess the substrate’s contribution to community structure. The first two component axes explained 100% of the total variance in the data. At the level of class, bacteria were divided into three completely independent microbiomes. These results indicated the microbiome strongly depended on the type of carbon applied for the enrichment process; the preference or dependence on substrate was the decisive factor driving community structure.

Our study showed that the dominant bacteria and PHA storing bacteria in the three enrichments were distinctive. Therefore, the nature of the substrate determines the community composition and species of PHA storing bacteria, which subsequently affects final PHA production capacity. Because three microbial communities had a stable functional base, bacteria with similar characteristics could drive the organization of the strict population ecology. In this environment, a metastable balance is likely to exist between populations of species that are both competing for, and also co-metabolizing, influent substrate components and the metabolic intermediates.

Acetate was the most favorable substrate for PHA-storing bacteria such as *Pseudomonas* sp. and *Stappia* sp.; this may explain their high relative abundance in the acetate-enriched MMC. The data also indicated other acetate-preferable bacteria in the phylum *Proteobacteria* could potentially compete for the substrate. Glucose was the most popular substrate for bacteria, and the community had a fairly evenly distributed bacterial species. Although these bacteria were affiliated with four phyla, they survived and were strongly competitive in the glucose-enriched MMC.

Starch is very different from the more easily degradable acetate and glucose, and had to be first hydrolyzed into an easily degradable material to be available to other microorganisms. This led to a close cooperative relationship between the bacteria and the starch substrate. The starch-enriched MMC was dominated by *Candidate_division_TM7* phylum, suggesting these organisms play a key role in the hydrolysis of starch. Bacteria in phylum of *Proteobacteria* and *Bacteroidetes* could not use starch directly, and were consequently regulated by the availability of substrate formed from starch hydrolysis. This bacterial hierarchy was highlighted in the starch-enriched MMC.

Analyzing the diversity of the three MMCs led us to conclude that a diverse microbial population does not impede the high functional specialization of PHA storage. Diversity resulted from substrate availability and preference, because these factors support more competition between species, expanding the ecological niche for multiple populations.

## Methods

### Enriching procedure

The inoculated seed was collected from the Tang River estuary in Qinhuangdao city of Hebei Province in China. The sampling and elutriation procedure was based on a previous study^34^. After elutriation, the inoculum was divided evenly into three double-jacket glass SBRs with a working volume of 2 L each. Compressed air was supplied through diffusers at the bottom of the reactors; controllers controlled the air flows. The reactors were concurrently stirred using magnetic stirrers to ensure sufficient oxygen transfer. The temperature was controlled at 30 ± 0.5 °C using a water jacket and a thermostat bath (SDC-6, China). All the reactors were operated using the same scheme.

The operational cycle consisted of 5-min feeding, 690-min aeration, 2-min sludge withdrawal, 20-min settling, and 3-min water withdrawal. The sludge residence time (SRT) was 5 d, and the hydraulic residence time (HRT) was 24 h. The reactors were purged once a week to remove biofilm from the reactor wall. The enrichment feedstock (referred to as feedstock 1) was compounded separately using sodium acetate, glucose, or starch as the single carbon. The carbon concentration in feedstock 1 for the three reactors was controlled equally based on COD measurement, at approximately 1400 mg∙L^−1^. The nutrient solution and trace elements in feedstock 1 equally consisted of 103 mg∙L^−1^ NH_4_Cl, 26 mg∙L^−1^ KH_2_PO_4_, 7.6 mg∙L^−1^ KCl, 45 mg∙L^−1^ MgSO_4_, 3.0 mg∙L^−1^ FeSO_4_∙7H_2_O, 1.5 mg∙L^−1^ ZnSO_4_∙7H_2_O, 8.0 mg∙L^−1^ CaCl_2_∙2H_2_O, and 1.2 mg∙L^−1^ MnSO_4_∙H_2_O. In addition, 20 mg∙L^−1^ allylthiourea was also added to feedstock 1 to prevent nitrification.

### Pulse-fed experiments to maximize PHA production

The enrichments collected from three SBRs were tested to assess maximum PHA production using pulse-fed experiments at the end of enrichment period. This process adopted the same setups as culture selection. The feedstocks for the pulse-fed experiments (referred to feedstock 2) had the same nutrient solution and trace element composition and concentration as feedstock 1, except no NH_4_Cl was added. This was done to inhibit growth and enhance PHA storage. Separate concentrated carbon feedstocks were prepared using acetate sodium, glucose, or starch, at the same concentration of 6 mol C∙L^−1^.

The inoculated biomass from the enrichment step was controlled at a mixed liquor suspended solids (MLSS) level of 3000 mg∙L^−1^. The feedstock 2 was filled into the 2 L SBRs. For these experiments, a pulse of 0.03 mol concentrated carbon solution was regularly added into the reactors. The pulse addition of the carbon source was done according to the DO signal (explained further in the section of PHA storing ability of enrichments using three kinds of substrates). The pulse-fed experiments continued until the PHA content in the biomass reached its maximum level.

### Calculations

Kinetic and stoichiometric analyses were carried out based on three continuous cycle measurements. The units of all organic matter concentrations were unified to a mg COD∙L^−1^ basis to facilitate calculations and comparisons. The initial active biomass was estimated by subtracting the initial PHA and ash mass from the initial VSS mass. This measure was then converted to the COD unit with a factor of 1.42 mg COD ∙mg biomass^−1^. This assumed that the cell formulation is C_5_H_7_O_2_N^35^.

Based on the same assumption, the subsequent incremental active biomass can be estimated using ammonium nitrogen consumption, at a factor of 11.43 mg COD∙mg N^−1^. The units of PHB and PHV measured in this study were converted to COD based on 1.38 mg COD∙mg PHB^−1^ and 1.63 mg COD∙mg PHV^−1^ reported by Dionisi *et al*.^5^. Using the kinetic model developed by Johnson *et al*.^36^, the substrate uptake can be expressed using the regular saturation kinetics in Eq. (1).


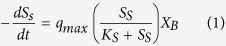


Likewise, biomass growth can be described using a Monod-type relation in Eq. (2).


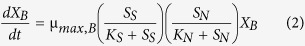


The PHA synthesis rate results from substrate uptake minus the substrate required for growth and maintenance purposes, as expressed in Eq. (3).


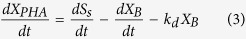


The decay rate (*k*_d_) measures the energy demand needed for cell maintenance, including functions such as repair, synthesis, osmotic regulation transport, and heat loss. As defined in standard nomenclature, the kinetic parameters, such as *μ*, *q*_S_, and *q*_PHA_, can be expressed as:


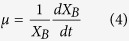



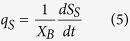



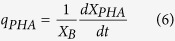


The kinetic model expressed in Eqs (4) to (6), 5, 6 was solved using Matlab-Simulink (Version R2012b), using the Simulink Design Optimization package to optimize parameters. The average observed storage yield (*Y*_PHA/S_) and average observed growth yield (*Y*_X/S_) were also calculated using Eqs (7) and (8).


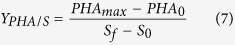



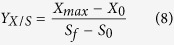


Here, PHA_0_, *X*_0_ and *S*_f_ refer to PHA content, biomass content, and substrate content at the start of the experiments, respectively. PHA_*max*_ refers to the maximum PHA content.

The Simpson index of community was calculated as Eqs (9).


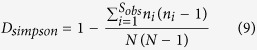


Here, *S*_obs_, *n*_i_ and *N* refer to the number of observed operational taxonomic units (OTU), the OTUs including *i* sequences, and all of the OTUs, respectively.

### Analysis Methods

Dissolved oxygen (DO) concentration, temperature, and pH were monitored using an online WTW Multi 340i Meter (WTW Company, Germany). Salinity was measured using a salinity meter (GMK510, Korea). Ammonium, MLSS, and the volatile mixed liquor suspended solids (MLVSS) were measured and analyzed using standard methods^37^. Samples collected for PHB/PHBV analysis were added to 50 mL tubes containing 1 mL of sodium hypochlorite to stop all biological activity. Samples were subsequently washed with tap water and freeze-dried. The PHB/PHBV content of each sample was determined using the method described by Oehmen *et al*. with gas chromatography (Agilent 7890A, USA)^38^.

### Microbial diversity analysis

To determine microbial diversity, mixed liquids were collected from each SBR at the 350th cycle. After 3-min centrifugation operating at 4500 rpm∙min^−1^, the liquid supernatant was discarded. The samples were then washed twice with tap water and then centrifuged twice to remove impurities. The purged sludge was freeze-dried at −20 °C, and the freeze-dried sludge samples were saved at −20 °C.

Microbial DNA was extracted from samples using a E.Z.N.A.^®^ Solid DNA Kit (Omega Bio-tek, Norcross, GA, U.S.), using manufacturer protocols. The V3+V4 regions of the bacteria 16S ribosomal RNA gene were amplified with PCR (95 °C for 2 min, followed by 25 cycles at 95 °C for 30 s, 55 °C for 30 s, and 72 °C for 30 s, and a final period at 72 °C for 5 min) using primers 338F 5′-ACTCCTACGGGAGGCAGCA-3′ and 806R 5′-GGACTACHVGGGTWTCTAAT-3′. This barcode is an eight-base sequence unique to each sample.

PCR reactions were performed using a Gene Amp PCR-System^®^ 9700 (Applied Biosystems, Foster City, CA, USA) in triplicate using a 20 μL mixture containing 4 μL of 5 × FastPfu Buffer, 2 μL of 2.5 mM dNTPs (TransStart Fastpfu DNA Polymerase, TransGen AP221-02, Beijing, China), 0.8 μL of each primer (5 μM), 0.4 μL of FastPfu Polymerase, and 10 ng of template DNA. Amplicons were extracted from 2% agarose gels and purified using the AxyPrep DNA Gel Extraction Kit (Axygen Biosciences, Union City, CA, USA) using manufacturer instructions and quantified using QuantiFluor™-ST (Promega, USA). Purified amplicons were pooled in equimolar and paired-end sequences (2 × 250) on an Illumina MiSeq platform, based on standard protocols.

Representative reads were deposited into the NCBI Genebank database (Accession Number: KP681274-KP681545). Raw fastq files were demultiplexed and quality-filtered using QIIME (version 1.17) with the following criteria: (1) The 250 bp reads were truncated at any site receiving an average quality score <20 over a 10 bp sliding window, discarding the truncated reads that were shorter than 50 bp. (2) Exact barcode matching, 2 nucleotide mismatch in primer matching, reads containing ambiguous characters were removed. (3) Only sequences that overlap longer than 10 bp were assembled based on their overlap sequence. Reads which could not be assembled were discarded.

OTUs were clustered using a 97% similarity using UPARSE (version 7.1 http://drive5.com/uparse/); chimeric sequences were identified and removed using UCHIME. Rarefaction data, Shannon, Richness and Chao1 indices were created using the Mothur program. The phylogenetic affiliation of each 16S rRNA gene sequence was analyzed by RDP Classifier (http://rdp.cme.msu.edu/) against the silva (SSU115)16S rRNA database using a confidence threshold of 70%. Functional gene and phylogenetic diversity indices and PCA were calculated using R software (version 2.15.1, http://www.r-project.org/).

## Additional Information

**How to cite this article**: Cui, Y.-W. *et al*. Effects of carbon sources on the enrichment of halophilic polyhydroxyalkanoate-storing mixed microbial culture in an aerobic dynamic feeding process. *Sci. Rep.* **6**, 30766; doi: 10.1038/srep30766 (2016).

## Supplementary Material

Supplementary Information

## Figures and Tables

**Table 1:** Kinetics analysis of the three enriched MMCs.

Fed carbon source	*μ*_*max*_ *h*^−1^	*K*_*s*_*mgCOD* *L*^−1^	*k*_*d*_*h*^−1^	*q*_*s,max*_*h*^−1^	*q*_*PHA,max*_*h*^−1^	*Y*_PHA/S_	*Y*_X/S_	reference
Acetic sodium	0.082 ± 0.003	86.46 ± 0.68	0.0569 ± 0.0021	0.461 ± 0.022	0.292 ± 0.015	0.60	0.24	
Glucose	0.082 ± 0.004	75.43 ± 1.66	0.0415 ± 0.0022	0.446 ± 0.017	0.148 ± 0.011	0.54	0.24	
Starch	0.023 ± 0.004	61.98 ± 2.29	0.1105 ± 0.0025	0.170 ± 0.023	0.032 ± 0.031	0.30	0.30	
Acetic sodium	—	—	—	0.361–0.655	0.156–0.327	0.47–0.59	—	Martins *et al*.
Acetic, lactic, propionic acids	—	—	—	0.68	—	0.41	—	Dionisi *et al*.
Crude glycerol	—	—	—	0.27	0.039	0.20	0.11	Moita *et al*.

“—” indicates no data available.

**Table 2:** Bacterial community diversity analysis sampled with different carbon sources.

Sample	Ace	Chao	Simpson	Evenness
Seeded sludge	211	212	0.97	0.81
Acetate-fed	45	44	0.86	0.66
Glucose-fed	80	79	0.91	0.67
Starch-fed	45	45	0.55	0.32

Note: The OTUs were defined by a 97% similarity.
