# Supplementary material for: Effects of carbon sources on the enrichment of halophilic polyhydroxyalkanoate-storing mixed microbial culture in an aerobic dynamic feeding process
Source: Sci Rep. 2016 Aug 3;6:30766. doi: 10.1038/srep30766 (PMC4971467; doi:10.1038/srep30766)
Supplement: Supplementary Information [file srep30766-s1.pdf]

Effects of carbon sources on the enrichment of halophilic polyhydroxyalkanoate-storing mixed microbial culture in an aerobic dynamic feeding process

You-Wei Cui*, Hong-Yu Zhang, Peng-Fei Lu, Yong-Zhen Peng

Beijing University of Technology, College of Energy and Environmental Engineering, 100 Pingleyuan, Chaoyang District, Beijing 100124, China

* corresponding. cyw@bjut.edu.cn

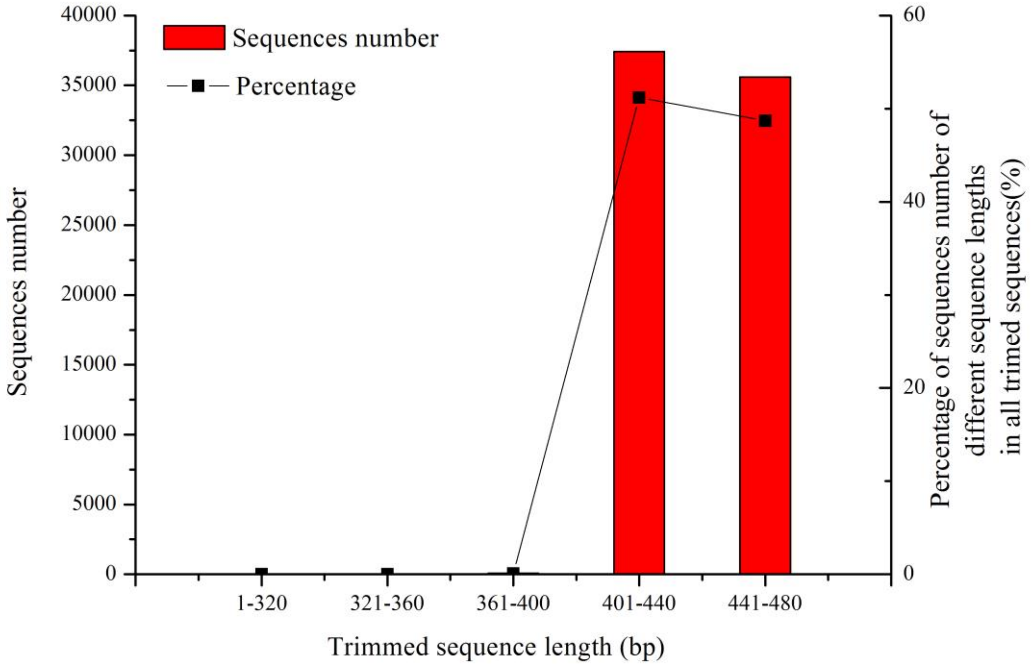

Figure S1. Sequence distribution

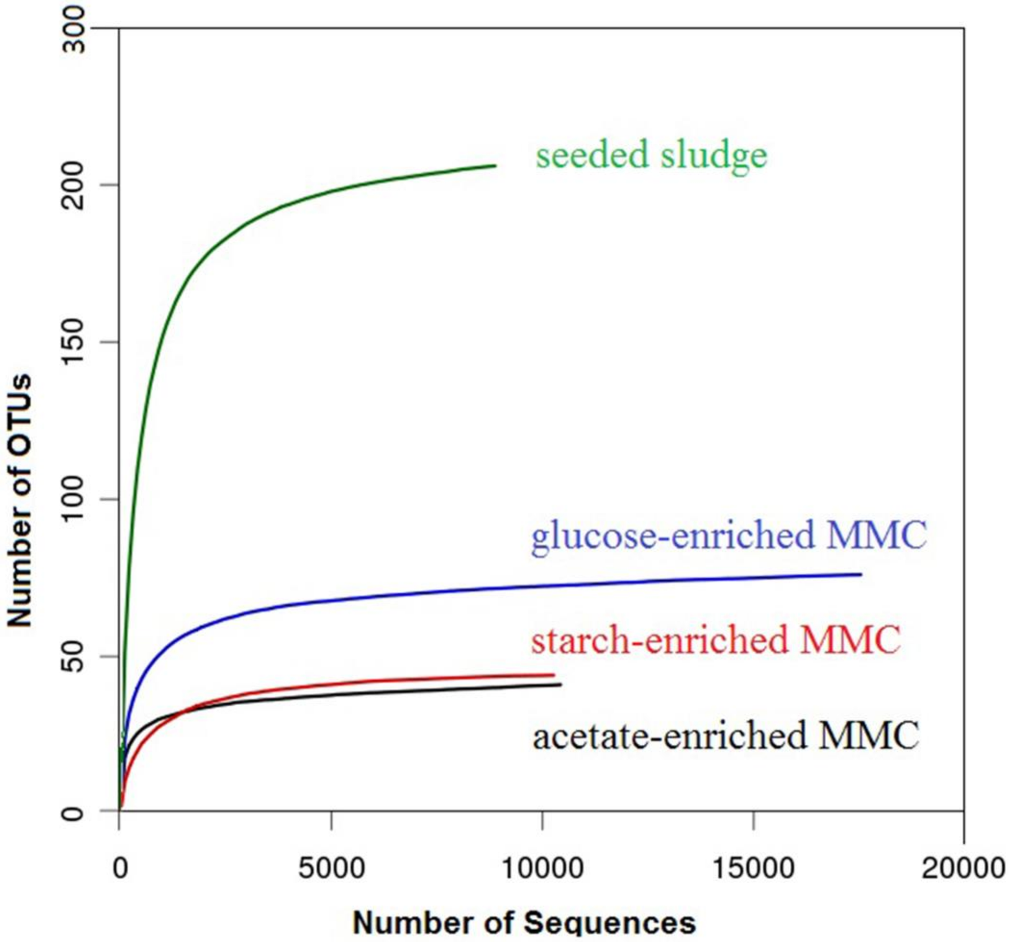

Figure S2. Rarefaction curve of four samples

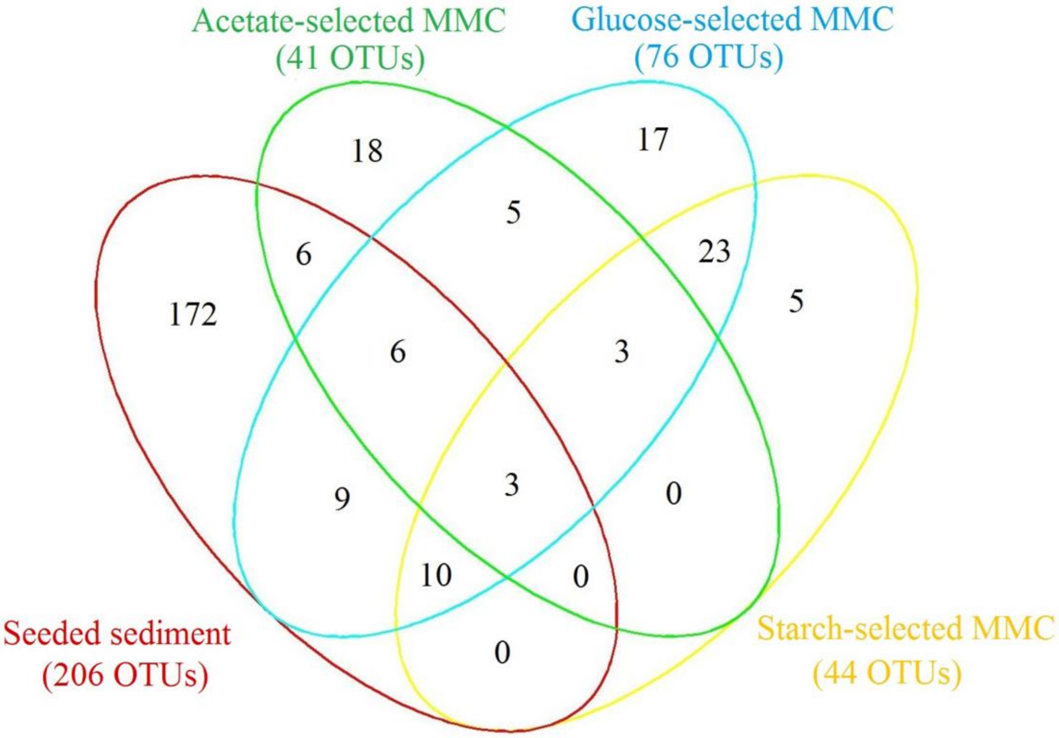

Figure. S3 Venn figure based on OTUs

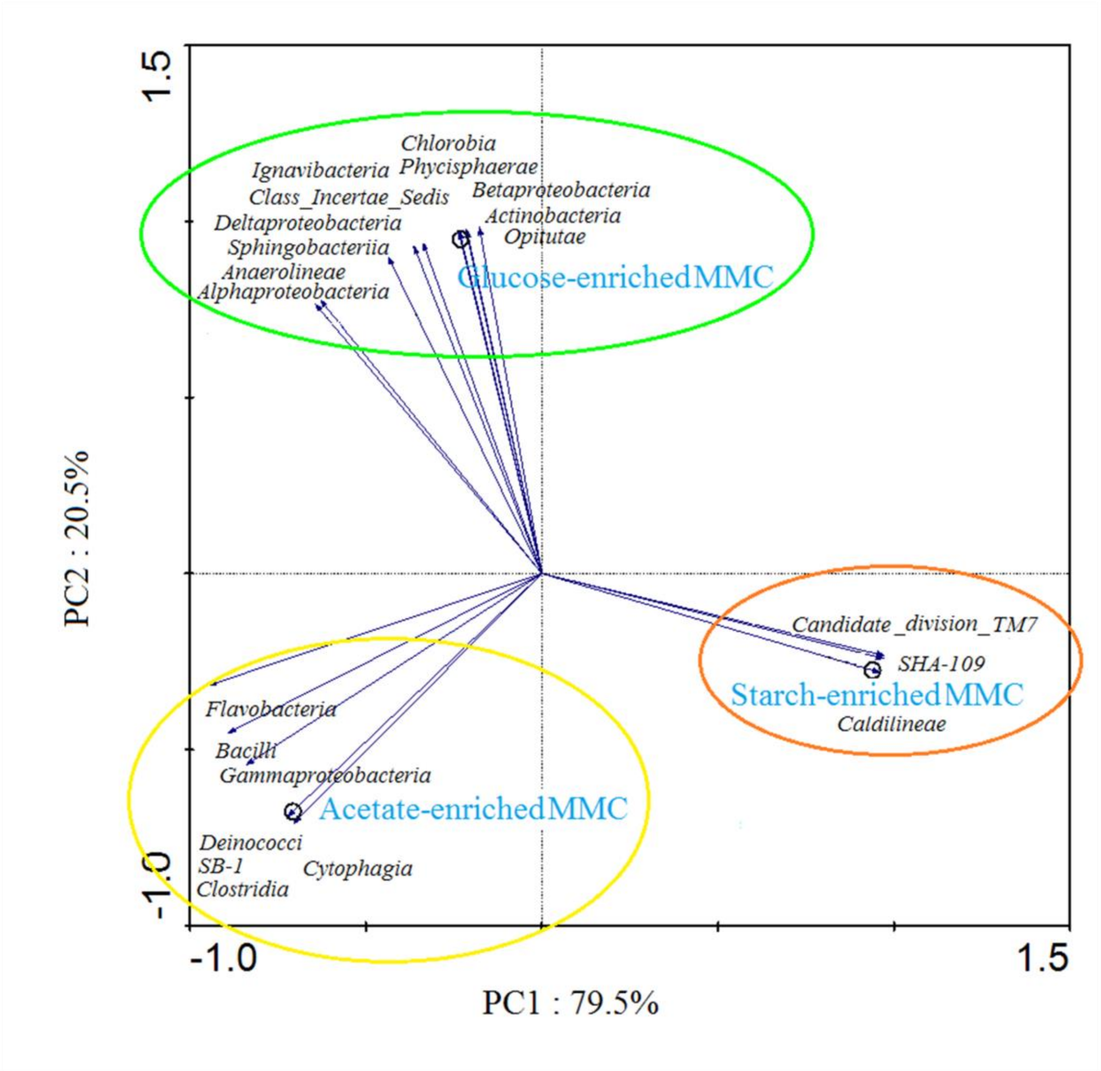

Figure S4. Relationship between genera and the three enriched communities based on PCA analysis
